# Surgery of the Heart and Lungs

**Published:** 1904-09

**Authors:** 


SURGERY OF THE HEART AND LUNGS. By Benjamin Merrell Ricketts, M. D., Ph. B., Member American Medical Association; Western Surgical and Gynecological Association; International Medical Congress, 1887; International Association Railway Surgeons; Mississippi Valley Medical Association; Cincinnati Academy of Medicine; Ohio State Medical Association; American Proctologic Society; Honorary Member Medical Society State of New York; Honorary Member St. Louis Medical Society; Fellow New York State Medical Association, and Member *Societi de Chirurgerie.* The Grafton Press, New York, 1904, Cloth, 500 pages, price $5; Half leather, $7.

This splendid work is a "history and résumé of surgical conditions found" in the heart and lungs, and "experimental and clinical research in man and lower animals, with reference to pneumonotomy, pneumonectomy, and bronchotomy, and cardiotomy and cardiorrhaphy." It shows deep and extensive research and experience and accurate observation. The book is copiously illustrated with very good cuts, which will greatly elucidate the text. It will be a valuable acquisition to any physician's possessions, and is very cheap at $5.

